# Acute Pectoralis Major Rupture Captured on Video

**DOI:** 10.1155/2016/2482189

**Published:** 2016-08-10

**Authors:** Alejandro Ordas Bayon, Enrique Sandoval, María Valencia Mora

**Affiliations:** ^1^Department of Orthopedic Surgery, Hospital Universitario Severo Ochoa, Avenida de Orellana SN, 28914 Leganés, Spain; ^2^Department of Orthopedic Surgery, Hospital Universitario Fundación Jiménez Díaz, Avenida Reyes Católicos 2, 28040 Madrid, Spain

## Abstract

Pectoralis major (PM) ruptures are uncommon injuries, although they are becoming more frequent. We report a case of a PM rupture in a young male who presented with axillar pain and absence of the anterior axillary fold after he perceived a snap while lifting 200 kg in the bench press. Diagnosis of PM rupture was suspected clinically and confirmed with imaging studies. The patient was treated surgically, reinserting the tendon to the humerus with suture anchors. One-year follow-up showed excellent results. The patient was recording his training on video, so we can observe in detail the most common mechanism of injury of PM rupture.

## 1. Introduction

PM ruptures are rare. Their incidence is rising due to the great number of weight-training injuries. They affect almost exclusively men aged between 20 and 40 years and, in some cases, they are associated with the use of anabolic steroids [1].

It is exceptional to capture on video the exact moment of PM rupture. To our knowledge, there are no videos showing the PM rupture during the eccentric phase of the bench press, which represents the most common mechanism of injury.

We present a typical acute case of PM rupture, surgically treated, with excellent final outcomes.

## 2. Case Presentation

A 29-year-old male presented with pain in the left axillary area and ecchymosis preceded by a snap while lifting 200 kg in bench press three days earlier. He admitted a previous history of anabolic steroid use, with the last consumption being six months earlier.

Physical examination revealed an extensive hematoma and swelling in the medial side of the left upper arm and absence of the anterior axillary fold with pain in that area. Shoulder range of motion, both passive and active, was complete.

Patient had recorded the training exercise so we could observe the injury. He was laying down on the bench press with an assistant by his head, and he was being recorded from his left side. He was lifting exactly 212.5 kg, and during the third repetition, at the beginning of the eccentric phase, the loss of the natural contour and immediate medial retraction of the PM muscle can be observed (video 1 in Supplementary Material available online at http://dx.doi.org/10.1155/2016/2482189).

Plain radiographs did not show any abnormality. To determine the extension and localization of the rupture, ultrasound (US) and magnetic resonance imaging (MRI) studies were performed. US were interpreted as a probable partial tear of the left PM tendon, while MRI reported on a rupture of PM muscle at myotendinous junction with medial retraction of the inferior portion of the muscle belly.

The patient was treated surgically, thirteen days after the injury. Under general anesthesia on a beach chair position, we performed a modified deltopectoral approach. A rupture affecting the musculotendinous junction was confirmed intraoperatively. The medial stump was identified and controlled with a total of three threads in a Krackow fashion from three corresponding suture anchors. Three holes were drilled lateral to the bicipital groove just where the native footprint was located. The three threads were firmly tightened passing the sutures through the drilled holes with the arm adducted and tied with simple knots.

The patient was postoperatively immobilized in a sling. The second week after surgery, he was allowed to start a passive range of motion; on the third month, he started with resisted motion exercises. On the fourth month, he had returned back to his normal physical activity. In the last follow-up, one year after surgery, he did not mention any pain and was satisfied with the aesthetic. The range of motion regarding the affected shoulder was normal and he had started performing some weightlifting, nevertheless not lifting so much weight as before.

## 3. Discussion

There are about four hundred cases reported in the literature of PM ruptures. Most of them belong to the last decade, which suggests that PM injuries and those associated with weightlifting are becoming more frequent, in relation to the significant increase in weight-training injuries reported in the last twenty years [1–5] and probably with a concurrent increment in anabolic steroid use [6].

Most cases occur in young active men [2], probably due to the lower elasticity of male tendons, lower tendon to muscle diameter, and an apparent affinity of male for high-energy activities; however, this has not been demonstrated [7].

Anatomically, PM muscle has a triangular-like shape and it origins in the medial clavicle, anterior sternum, first to sixth costal cartilage, and aponeurosis of the oblique external muscle of the abdomen. Its muscular belly has two heads or portions: a clavicular head and a sternal head. The sternal head is also subdivided into another seven segments, although they are not constant. Both heads converge in short, wide, flattened, and bifascicular tendon inserting in the humerus, lateral to the bicipital groove. The two fascicles or layers of the tendon are one anterior, formed by the clavicular head and the more superior segments of the sternal head, and one posterior, formed by the inferior segments of the sternal head. It is remarkable that the clavicular head is shorter than any other of the sternal segments but the two last two segments, S6 and S7, are about 1 to 2 cm shorter than the segments above and the angle of lateral attachment is greater also in these two segments [8, 9].

The main function of the PM muscle is to adduct and internally rotate the shoulder, although it also participates in flexion through the clavicular head [10]. It is a powerful muscle, highly developed in athletes, and its rupture has been related with anabolic steroid use [7, 9, 11].

The most common mechanism of injury is an indirect trauma, during the eccentric phase of the bench press exercise, when the shoulder is abducted, extended, and externally rotated [2, 3, 12]. In this position, PM disrupts in a predictable sequence, being the most inferior segments of the sternal head the first to fail, due to the relative shorter length and greater lateral attachment angle, which generates bigger tensions. The most superior segments of the sternal head and the clavicular head follow the disruption [10]. This can be observed in our video.

Pochini et al. [13] reported on a rupture of pectoralis major captured on video, occurring between the transition between eccentric and concentric phases during a bench press contest in a powerlifting athlete, who also had an anabolic steroid consumption history. It is probable that steroid use leads to abnormal muscle hypertrophy and tendinopathy [14] and thus rupture happens in concentric phase when lifting extremely high weight.

Diagnosis is based on a compatible history and physical examination [3, 15, 16]. Findings as pain in the medial side of the upper arm, swelling and ecchymosis, asymmetry, and weakness with adduction and internal rotation are common, but the most useful sign is the absence of the anterior axillary fold evidenced by resisted adduction or passive abduction of the affected arm [12].

Simple X-rays must be taken to rule out the infrequent cases of bony avulsion. MRI is the preferred imaging technique to determine rupture extension and localization [15]. US requires a more experienced operator, although it can be used if the diagnosis is not clear or when there is an unacceptable delay to MRI [12].

Nowadays the classification system proposed by ElMaraghy and Devereaux [1] is the most complete one. It includes injury timing, acute versus chronic; location, muscle, tendon, bony, avulsion; and extension, width and thickness.

Treatment options vary, depending on patient and injury type. Factors that must be considered are pain, range of motion, adduction weakness or power decrease, aesthetical defect, occupation, and activity level [2, 7, 11, 17]. Conservative treatment is limited to low-demanding patients and partial-tendon or muscle-fibers ruptures [18]. It consists of immobilization, analgesics, ice, and physiotherapy [12]. A prospective study, level 2 of evidence, stated the poor outcomes of conservative treatment in athletes [19].

Many surgical techniques have been described to repair PM ruptures, including tendon-to-tendon suture, bone trough repair, anchor sutures, transosseous sutures, and tendon reinsertion to clavipectoral fascia [20, 21]. In most cases, direct repair to bone is possible with either a transosseous or a suture-anchor repair [15]. Early surgical treatment has demonstrated better outcomes than conservative [3, 7, 18, 22], especially in active patients [11, 17–19, 23]. A meticulous surgical technique and specific rehabilitation programs have been shown to play a more important role in outcomes than a delay in surgery [15, 16, 24, 25]. In case of chronic ruptures, autografts or allografts may be necessary [15]. Surgical complications include infection, heterotopic ossification, injury to the long head of the biceps, neurovascular injuries, and rerupture (0–7.7%) [12]. In an experimental study [26], the majority of failures occurred through the suture used for tendon repair, especially regarding suture-anchor repairs compared with bone trough group.

Postoperatively an individualized rehabilitation protocol is essential. Immediately after surgery, immobilization with sling allowing only shoulder passive motion until the third-fourth week is recommended [22]. Active assisted motion is started between third and sixth weeks, progressing then to active motion [15]. Shoulder is protected from normal life activities for four to six months postoperatively.

## 4. Conclusions

Pectoralis major ruptures occur more frequently during the eccentric phase of bench press in young male adults because, in this position, the more inferior segments of the muscle are overloaded. Although videos of PM ruptures during eccentric phase can be found, they are not published in recognized medical literature. Our purpose of this paper is to provide access to it to health care professionals as well as bringing a small and helpful review of anatomy, diagnosis, imaging, treatment possibilities, and postoperative management.

## Supplementary Material

The following video shows the moment of PM rupture in the eccentric phase of the bench press while lifting 212.5 kg.
